# Sampled ensemble neutrality as a feature to classify potential structured RNAs

**DOI:** 10.1186/s12864-014-1203-8

**Published:** 2015-02-05

**Authors:** Shermin Pei, Jon S Anthony, Michelle M Meyer

**Affiliations:** Boston College, 140 Commonwealth Ave., Chestnut Hill, 02467 MA USA

**Keywords:** RNA structural robustness, RNA de novo discovery, RNA structural ensemble, Mutational robustness

## Abstract

**Background:**

Structured RNAs have many biological functions ranging from catalysis of chemical reactions to gene regulation. Yet, many homologous structured RNAs display most of their conservation at the secondary or tertiary structure level. As a result, strategies for structured RNA discovery rely heavily on identification of sequences sharing a common stable secondary structure. However, correctly distinguishing structured RNAs from surrounding genomic sequence remains challenging, especially during *de novo* discovery. RNA also has a long history as a computational model for evolution due to the direct link between genotype (sequence) and phenotype (structure). From these studies it is clear that evolved RNA structures, like protein structures, can be considered robust to point mutations. In this context, an RNA sequence is considered robust if its neutrality (extent to which single mutant neighbors maintain the same secondary structure) is greater than that expected for an artificial sequence with the same minimum free energy structure.

**Results:**

In this work, we bring concepts from evolutionary biology to bear on the structured RNA *de novo* discovery process. We hypothesize that alignments corresponding to structured RNAs should consist of neutral sequences. We evaluate several measures of neutrality for their ability to distinguish between alignments of structured RNA sequences drawn from Rfam and various decoy alignments. We also introduce a new measure of RNA structural neutrality, the structure ensemble neutrality (SEN). SEN seeks to increase the biological relevance of existing neutrality measures in two ways. First, it uses information from an alignment of homologous sequences to identify a conserved biologically relevant structure for comparison. Second, it only counts base-pairs of the original structure that are absent in the comparison structure and does not penalize the formation of additional base-pairs.

**Conclusion:**

We find that several measures of neutrality are effective at separating structured RNAs from decoy sequences, including both shuffled alignments and flanking genomic sequence. Furthermore, as an independent feature classifier to identify structured RNAs, SEN yields comparable performance to current approaches that consider a variety of features including stability and sequence identity. Finally, SEN outperforms other measures of neutrality at detecting mutational robustness in bacterial regulatory RNA structures.

**Electronic supplementary material:**

The online version of this article (doi:10.1186/s12864-014-1203-8) contains supplementary material, which is available to authorized users.

## Background

RNA plays key roles in both bacterial and eukaryotic gene regulation [1,2], and changes to RNA structure have been implicated as causes for human genetic diseases [3]. Yet, unlike protein sequences which are readily identified in genomic sequences, RNAs with homologous functions may be difficult to identify in genomic sequences due to a lack of well defined start and stop signals and poor primary sequence identity [4,5]. Rather, the biological function of structured RNAs often depends on a well-defined three-dimensional shape that is largely determined by interactions between discrete and stable secondary structure elements [6-8]. These structural constraints lead to covarying mutations, a conservation pattern characterized by the maintenance of base-pairing interactions involved in RNA secondary structure [9,10]. These features are exploited to identify homologous sequences of previously characterized structured RNAs and to discover new putative RNAs [11]. However, this process is often further complicated by the potential for multiple biologically functional conformations [12], and cases where only a portion of a larger RNA structure is required for biological function. For example, RNAse P is a ribozyme involved in the maturation of small noncoding RNAs whose phylogentically conserved core is functional in isolation, although with significantly decreased activity [13,14]. Despite these challenges, several computational tools have been developed both for RNA homology searching and *de novo* structured RNA identification [11,15].

*De novo* non-coding RNA (ncRNA) discovery in genomic sequence is largely accomplished with computational tools that identify a stable thermodynamic structure that is maintained across many species [16-19]. While thermodynamic stability alone is not sufficient to distinguish functional structured RNAs from random genomic sequence [20], the rapid growth of sequence databases has allowed the use of comparative genomics to determine whether such putative stable structures are conserved, and to identify the characteristic covarying mutation pattern of structure conservation within predicted pairing elements [11]. Machine learning techniques, specifically support vector machines (SVMs) [17,19], leverage both the thermodynamic stability of structured RNA, and the presence of covarying mutations as an indicator of conserved structure, to distinguish alignments corresponding to putative biologically functional structured RNAs from alignments of sequences conserved for other reasons and non-conserved thermodynamically stable structures. There are six quantifiable features commonly used by *de novo* ncRNA prediction approaches including: the thermodynamic stability of the structures formed by individual sequences, as measured by the mean of the Z-score of the minimum free energy (MFE) structure of sequences in a putative alignment [21,22]. The ability of sequences in the alignment to fold into the common predicted consensus structure, as measured by the structure conservation index [17]. The extent to which sequences are diverse and contain covarying mutations, as measured by the mutual information [9], entropy [23] of base-pairing regions, and the mean pairwise sequence identity between alignment sequences. Finally, because more sequences lead to higher prediction accuracy, the number of sequences in the alignment is also a common feature.

There exists a facile computational link between RNA sequence and secondary structure due to the considerable efforts toward RNA secondary structure prediction. As a result, simulation of RNA evolution using structure as a proxy for fitness has been used to explore a variety of evolutionary ideas [24-26]. These studies have shown that sequences with the same structure often are part of networks of sequences separated by single mutations (1-mutant neighbors) that share an MFE structure [4,27]. *In silico* experiments reveal that some structures are mutationally robust because they have large networks of highly connected sequences [28] allowing them to maintain structure while tolerating many different mutations. Using *in silico* methods, mutational robustness has been demonstrated for naturally occurring RNAs such as pre-miRNAs [29] and virus genome elements [30], though RNAs without structure (e.g. sRNAs) do not seem to display this feature [31,32].

Mutational robustness, therefore, should be a feature that can distinguish between random putative structures formed by genomic sequence, and biologically relevant ncRNA structures. Robustness is measured using neutrality, which is calculated as the mean secondary structure similarity (i.e. normalized base-pair distance) between a sequence and those that differ by exactly one point mutation (1-mutant neighbors) [29]. There are a variety of existing computational methods [33] and programs designed to evaluate RNA robustness (e.g. RNAmute, RDMAS, RSRE, RNAmutants, SNPfold, RNAsnp, RemuRNA, and Rchange) [3,5,34-39]. All of these approaches focus on a single input sequence and the ability of its neighboring mutants to maintain a “wild-type” structure. RNAmute, RSRE, and RDMAS evaluate the normalized base-pair distance between an MFE starting structure and the low energy suboptimal structures generated for mutant sequences using the Vienna RNA package [34,35,39]. However, using the MFE structure as the sole reference limits the accuracy of predicted structure disrupting mutations [40]. RNAmutants samples mutant sequences and structures according to their probability in the structural ensemble to identify sequences that severely disrupt structure, but fundamentally determines the structural disruption based on the MFE structure of the mutant [36]. To improve the accuracy of structure comparisons, SNPfold compares the structure ensemble of an RNA sequence with that of its mutants using the Pearson’s correlation coefficient (PCC) [3], and RNAsnp uses this measure in combination with the base-pair distance to evaluate structural similarity and disruption [37]. RemuRNA measures the effect of a mutation on the entire RNA secondary structure distribution using relative entropy rather than sampling from the structural ensemble [5]. Alternatively, Rchange takes a different approach and reports the expected change in mean ensemble free energy and thermodynamic entropy of structures [38].

In this work, we propose utilizing sequence neutrality as an SVM feature to classify potential structured RNAs. To do so, we introduce a new measure of neutrality, the structural ensemble neutrality (SEN). Similar to previous efforts to assess RNA robustness, this measure considers the thermodynamic ensemble of structures for 1-mutant neighbors and their difference from a given reference structure. However, rather than utilize the MFE structure of our initial sequence as the reference structure, we utilize a structure that is derived from a multiple sequence alignment (MSA) of homologous RNAs to more accurately reflect the biologically relevant structure [41]. In addition, to account for the over-prediction of secondary structure elements relative to tertiary structure interactions necessary for function, our distance metric prioritizes maintenance of the existing structure rather than considering all base-pair changes (both newly formed and broken base-pairs) as equal. We demonstrate that this measure of neutrality successfully distinguishes alignments of known bacterial structured regulatory RNAs from several different types of decoy data including both shuffled alignments and alignments constructed from intergenic or protein-coding sequence. We extend this finding to evaluate neutrality as a feature for classification of putative ncRNA alignments using an SVM. This analysis shows that neutrality can correctly classify ncRNA alignments nearly as well as the combination of existing features implying that the calculation of neutrality encompasses many of these existing features. Finally, we also show that many RNAs involved in bacterial regulation are mutationally robust using the structural ensemble neutrality.

## Methods

### Sequence neutrality

Before calculating neutrality, some common variables must be defined. Let a given input sequence *S*, without gaps and of length *L*, fold into a structure *T*. The set of sequences which differ from *S* by one point mutation are the 1-mutant neighbors

(1) $$ 1mut(S) = \{\text{1-mutant neighbors}\}  $$

Additionally, the size of the set 1*mut*(*S*) is |1*mut*(*S*)|=3*L*. A single 1-mutant neighbor of *S* is represented by *S*^′^ such that *S*^′^∈1*mut*(*S*). Let the structure ensemble of *S*^′^ be

(2) $$ e(S^{\prime}) = \{ \text{structure ensemble of } S^{\prime} \}  $$

The set of all *e*(*S*^′^) created from 1*mut*(*S*) is defined

(3) $$ \Gamma_{S} = \{e(S^{\prime}) | S^{\prime} \in 1mut(S) \}  $$

Additional functions using *e*(*S*^′^) are:

(4) $$ MFE(e(S^{\prime}))= \{ \text{the MFE structure of \(e(S^{\prime})\)} \}  $$

where |*MFE*(*e*(*S*^′^))|=1 and

(5) $$ T^{\prime}_{Nsamp}= \{sample(N,e(S^{\prime})) \}  $$

is created using RNAsubopt which samples *N* structures with replacement from *e*(*S*^′^) according to their probability of occurrence [42]. Let the secondary structure be represented as an *L*×*L* adjacency matrix *M* where an entry

(6) $$ M_{i,j} = \left\{ \begin{array}{l l} 1 & \text{if position}\; i \;\text{and}\; j \;\text{base pair}\\ 0 & \text{otherwise} \end{array} \right.  $$

The base-pair probability matrix for all base-pairs *i*,*j* in $T^{\prime }_{\textit {Nsamp}}$ is determined by calculating

(7) $$ BPROB(T^{\prime}_{Nsamp}) = \frac{1}{|T^{\prime}_{Nsamp}|} \sum\limits_{T^{\prime} \in T^{\prime}_{Nsamp}} M_{T^{\prime}}  $$

where $M_{T^{\prime }}$ is the adjacency matrix *M* for a sampled structure in $T^{\prime }_{\textit {Nsamp}}$. Alternatively, the base-pair probabilities can be explicitly calculated using ‘RNAfold -p’ in the Vienna RNA folding suite and parsing the resulting postscript file. However, we find this process to be be somewhat slower in aggregate. The centroid structure only represents base-pairs occurring in more than half of the sampled structures

(8) $$ \begin{aligned} cent(T^{\prime}_{Nsamp}) &= \left\{ \begin{array}{ll} 1 & \text{if}\,\,BPROB(T^{\prime}_{Nsamp})_{i,j} > 0.5\\ 0 & \text{otherwise} \end{array} \right.\\ & \forall \{i,j\} \in BPROB(T^{\prime}_{Nsamp}) \end{aligned}  $$

For some distance metric calculations, the secondary structure must be converted to a vector representation which represents a base-pairing character as 1 and 0 otherwise

(9) $$ \begin{aligned} V(structure) &= \left\{ \begin{array}{ll} 1 & \text{if position}\,\, i\,\, \text{is base-pairing}\\ 0 & \text{otherwise} \end{array} \right.\\ & \forall i \in structure \end{aligned}  $$

Neutrality calculations fundamentally rely on two factors: the accuracy of the two structures being compared (*T* and *MFE*(*e*(*S*^′^)) or $cent(T^{\prime }_{\textit {Nsamp}})$), and the distance metric used to measure the difference between these two structures. In this work, we develop a novel measurement of neutrality, the structural ensemble neutrality (SEN) and compare it with several existing neutrality measures. These include neutrality as determined by the programs RNAmute and RemuRNA. To allow direct comparison of different distance metrics we implemented the normalized base-pair distance (bp-distance), and the Pearson’s correlation coefficient (PCC). RNAmute takes a sequence *S* and reports neutrality. RemuRNA takes an input sequence (*S*) and calculates the Kullback-Leibler divergence (KLD) between *e*(*S*) and each *e*(1*mut*(*S*)). In our assessment, we take the mean KLD over all 1-mutant neighbors.

We implement normalized base-pair distance as

(10) $$ \frac{1}{|1mut(S)|} \sum \limits_{S^{\prime} \in 1mut(S)}{1- \frac{d(T,MFE(e(S^{\prime})))}{L}}  $$

where *d*(*T*,*MFE*(*e*(*S*^′^))) is the base-pair distance between the given structure *T* and the MFE structure of *S*^′^ (*MFE*(*e*(*S*^′^))) [29]. PCC is calculated by

(11) $$  \frac{1}{|1mut(S)|} \sum \limits_{S^{\prime} \in 1mut(S)}{\frac{1-d(V(T),V(cent(T^{\prime}_{Nsamp})))}{2}}  $$

where $d(V(T),V(cent(T^{\prime }_{\textit {Nsamp}})))$ is the Pearson’s correlation coefficient between the structure vector *V*(*T*) and the centroid structure vector $V(cent(T^{\prime }_{\textit {Nsamp}}))$ created from 1000 sampled structures of *e*(*S*^′^) [40].

Our novel neutrality measure, the structural ensemble neutrality (SEN), leverages two factors to increase the biological relevance of neutrality measurements. First, we focus on maintenance of the core RNA structure (i.e. minimal structure for biological function). Rather than consider all base-pair changes deleterious, only base-pairs in the original structure *T* disrupted in *T*^′^ are counted by our measurement. Second, we utilize a structure derived from comparative genomics as the reference structure *T* rather than the *MFE*(*e*(*S*)). This choice reflects understanding in the field that consensus structures defined from phylogenetic studies are much more likely to be accurate [43]. Structural ensemble neutrality is calculated by

(12) $$ \frac{1}{|1mut(S)|} \sum \limits_{S^{\prime} \in 1mut(S)}{\frac{1}{|T^{\prime}_{Nsamp}|} \sum \limits_{T^{\prime} \in T^{\prime}_{Nsamp}}{\frac{|T \cap T^{\prime}|}{|T|} }}  $$

*T*^′^ is a suboptimal structure of *S*^′^, |*T*| is the number of base-pairs in *T* and |*T*∩*T*^′^| is the number of base-pairs shared by both structures; therefore, $\frac {|T \cap T^{\prime }|}{|T|}$, a modification of Jacard distance, is the fraction of base-pairs in *T* retained in *T*^′^. To simplify equation 12, the distance measure comparing *T* to $T^{\prime }_{\textit {Nsamp}}$ is the mean fraction of bases retained

(13) $$ d(T,T^{\prime}_{Nsamp})= {\frac{1}{|T^{\prime}_{Nsamp}|} \sum \limits_{T^{\prime} \in T^{\prime}_{Nsamp}}{ \frac{|T \cap T^{\prime}|}{|T|} }}  $$

Here, $|T^{\prime }_{\textit {Nsamp}}|=1000$ because sampling 10000 structures does not significantly improve the results and sampling 100 structures causes inconsistent results due to small sample size. Substituting equation 13 into equation 12 results in

(14) $$  SEN= \frac{1}{|1mut(S)|} \sum \limits_{S^{\prime} \in 1mut(S)}{d\left(T,T^{\prime}_{Nsamp}\right)}  $$

### Alignment neutrality calculation

To streamline our process, we created a pipeline to calculate the neutrality of sequences in an MSA that can accommodate all neutrality measures in a uniform manner. This pipeline consists of a 3-step workflow. Starting with a structure alignment, 1) *S* and *T* are created by selecting a sequence and simultaneously degapping both the sequence and structure. In addition, structure positions with non-canonical base-pairings (not Watson-Crick or G-U wobble) are considered single stranded. 2) From *S*, we calculate 1*mut*(*S*) (Equation 1) and *Γ*_*S*_ (Equation 3). 3) Neutrality is calculated by utilizing the distance between the elements of *Γ*_*S*_ and *T*, which are calculated using a specified distance function: normalized base-pair distance (bp-distance) (Equation 10), Pearson’s correlation coefficient (PCC) (Equation 11), or sampled ensemble neutrality (SEN) (Equation 14).

### Test data

Data used to construct the test data sets was drawn from 35 seed alignments of regulatory structured RNAs found in bacteria (Additional file 1: Table S1) from the RNA Families database (Rfam) [44]. Regulatory RNAs in bacteria were chosen due to the large size and diversity of alignments available, as well as the structural data that verify many of the predicted structures. Several data sets were constructed by varying how the positive and negative alignments were generated. Positive alignments were generated by either utilizing all sequences in the Rfam seed alignment (all), or a randomly chosen subset of 3-6 sequences (subset). Structural information for these alignments was either derived directly from the RFam seed alignment (given) or calculated using RNAalifold (predicted) [45] (Table 1). For each positive data set, a corresponding set of negative training alignments were created using one of three methods: dinucleotide shuffle of the positive alignments (shuffled) [46], gathering 5’-flanking, or 3’-flanking, genomic sequence for each entry in the alignment (5’ and 3’ respectively). To control for sequence verse structure alignment, the 5’ and 3’-flanking sequences are aligned using ClustalW or Mxscarna [47]. All negative alignment consensus structures are calculated using RNAalifold [45].

**Table 1:** **Summary of data set sources**

**Data set**	**Sequence**	**Structure**	**Negatives**
1	subset	predicted	shuffled
2	all	given	3’,5’,shuffled
3	subset	predicted	3’,5’

### Impact of alignment quality on SEN

In order to assess the impact of alignment quality on SEN values, we determined the difference between SEN values obtained using an entire Rfam seed alignment (full alignments, positive Dataset2) or subsets of this alignment (subalignments, positive Dataset3). The delta SEN (SEN of full alignment - SEN of subalignment) is an estimate for the distance from the “true” SEN value obtained when using a subset of sequences that may result in a lower quality alignment and structure. To gauge how the delta SEN corresponds to differences between the structure predicted from the subalignment and the given structure from the Rfam alignment we examined the delta SEN as a function of two measures of structural difference: the bp-distance, and the ratio of the number of base pairs in the full alignment compared to the subalignment.

### Positional neutrality

Let $S^{\prime }_{i}$ be the set of three possible point mutations of *S* at a given position *i*.

(15) $$ S^{\prime}_{i} = \{ S^{\prime} \in 1mut(S) | S^{\prime} \text{contains point mutation at } i \}  $$

Positional neutrality is calculated by averaging equation 13 over $S^{\prime }_{i}$

(16) $$  SEN(i) = \frac{1}{|S^{\prime}_{i}|} \sum \limits_{S'_{i}} { d(T,T^{\prime}_{Nsamp}) }  $$

### Mutational robustness

For a sequence *S* to be considered mutationally robust, *neutrality*(*S*) must be greater than the mean background neutrality (i.e. inverse folded sequences). Mutational robustness of *S* is calculated by comparing its neutrality to the mean neutrality of 100 inverse folded sequences (Equation 17).

(17) $$ neutrality(S) > \frac{1}{100}\sum\limits_{i=1}^{100}neutrality(inv)_{i}  $$

For each sequence tested for robustness, RNAinverse [48] was used to generated 10 inverse folded sequences and each of those are used to seed 10 random walks resulting in a total of 100 inverse folded sequences for each *S*. Input sequences were omitted if no inverse folded sequence could be made from its structure.

RNAinverse [48] was used to generate an initial null set of sequences for comparison. As an alternative, we also used RNAifold to generate inverse folded sequences. However, the alignment consensus structure is not necessarily the MFE structure, which often causes RNAifold to fail and return no sequences. Because of this failure-mode, we did not force the inverse folded sequences to have the an MFE structure identical to the target structure when using RNAinverse. To control for base composition [29], the generated inverse folded sequences were constrained by Jensen-Shannon divergence (JSD) < 0.01 when compared to *S*. This process yielded an initial set of background sequences.

To ensure that background sequences generated by RNAinverse [48] are unbiased with respect to neutrality [49], the inverse folded sequences were used as a seed for a random walk along neutral sequences [31]. 4L steps are attempted and a step will be accepted only if the structure does not change. Any mutation which occurs in a base pair will also get a compensatory mutation to restore base pairing. If the random mutation results in the base being changed to a G, then the compensatory mutation will be randomly chosen, with equal probability, between a C and U.

### Support vector machine

To implement a binary classifier support vector machine (SVM), the LibSVM package [50] in R was used. The SVM uses the calculated features to classify an input sequence as either “structured RNA” or “other”. The features used are a standard 6-feature set, including the Z-score of the MFE, structure conservation index, mean entropy of stems, mean mutual information of stems, mean pairwise identity and number of sequences [17,19], and neutrality, which is calculated using the measures described above. Performance of the SVM is evaluated by using 10x cross-fold validation on a data set and compared by calculating the area under the curve (AUC) in receiver operating characteristic (ROC) curve analysis.

### Statistical analysis

All statistical tests were done in the R project for statistical computing. To test the significance of the separation of neutrality between structured and unstructured sequence, we used the Wilcoxon rank sum test, which is a non-parametric test and does not assume normally distributed data. Individual measures of neutrality were considered independently in this analysis.

To test correlation of neutrality using different measures, we first standardized the data by calculating the mean neutrality of RNA families because not all sequences are compatible with the structure or neutrality measure. Then the correlation was determined using the Spearman’s rank correlation coefficient.

Logistic regression was carried out using a generalized linear model where neutrality was used to predict the structure disruption, represented as 0 (no disruption) or 1 (disruption).

## Results and discussion

### Reference structure and distance metric impact calculated neutrality

**Figure 1:**
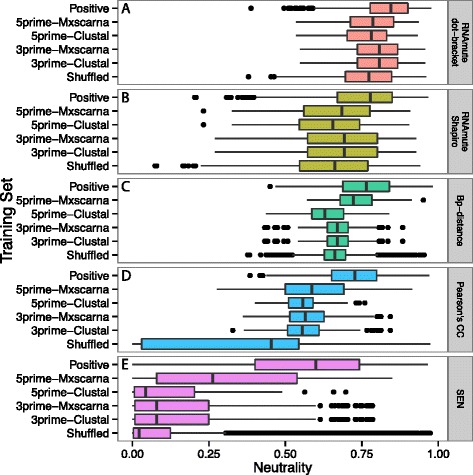
**SEN calculated neutrality has larger separation between structured and unstructured sequence.** Distribution of neutrality values from Dataset2 compare the performance of various distance functions **(A)** RNAmute dot-bracket representation, **(B)** RNAmute Shapiro representation, **(C)** bp-distance, **(D)** Pearson’s Correlation Coefficient (PCC), and **(E)** Sampled Ensemble Neutrality (SEN). The 3’ and 5’ flanking region used for negatives are referred to as 3prime and 5prime, respectively. The SEN on the positive test set has a larger separation between the negatives, compared to other measures. All distance metrics show unstructured sequence to be low on their respective scales. Lastly, the SEN uses a large dynamic range of values compared to the base pair distance metric which will increase its sensitivity between highly similar structures.

A set of structured RNA alignments derived from Rfam seed alignments (Dataset2, Table 1, Additional file 1: Table S1) was used to validate SEN as a measure of neutrality by comparing its performance to other measures that are the basis of most programs designed to capture RNA structural robustness: bp-distance and PCC. First, bp-distance performance was evaluated using both the original method which only takes an input sequence, implemented in RNAmute, and a modified version we implemented, which requires a given sequence and structure. By comparing these bp-distance implementations, we examine the effect of the input structure on neutrality and establish a baseline performance to compare SEN with existing methods. In addition, RNAmute can use two different structure representations to provide either a fine grained view (dot-bracket (db) notation) or coarse grained view (Shapiro representation) of structure to calculate base-pair distance. The neutrality RNAmute calculated using the db notation shows a small separation between structured (median = 0.8454) and unstructured sequences (medians = 5’-Clustal = 0.7807, 5’-Mxscarna = 0.7855, 3’-Clustal = 0.8069, 3’-Mxscarna = 0.8069, Shuffled = 0.7731) (Figure 1A). Using the Shapiro structure as an alternative representation to calculate neutrality shifted the neutrality lower (structured median = 0.7777, unstructured medians = 0.6553, 0.6850, 0.6925, 0.6925, 0.6615), but the results remain highly correlated (*ρ*=0.9306) (Table 2) with the db structure notation results (Figure 1B) indicating similar performance. However, using our modified version of bp-distance that imports the structure from the alignment does incrementally improve separation of structured RNAs and negative data (0.7654 vs. 0.6293, 0.7229, 0.6692, 0.6692, 0.6618) compared to RNAmute (Figure 1C) demonstrating that using the consensus structure from the alignment improves the accuracy of the structure. The correlation between using the MFE structure and a given structure (*ρ* = 0.565) indicates that using the given structure may improve the neutrality calculation but does not completely deviate from existing methods.

**Table 2:** **Spearman’s correlation between distance measures**

			**RNAmute**	
	**PCC**	**SEN**	**db**	**Shapiro**
Bp-distance	0.221	0.256	0.565	0.501
PCC		0.614	0.608	0.595
SEN			0.608	0.651
RNAmute-db				0.930

To assess alternative distance metrics, we compared SEN, PCC, and bp-distance. Using PCC to calculate neutrality shows a better separation between structured (median = 0.7369) and unstructured sequences (medians = 0.5569, 0.5857, 0.5555, 0.5649, 0.4535) than bp-distance (Figure 1D). Again the calculated neutrality is moderately similar to existing methods (*ρ*=0.608) indicating consistency with RNAmute. SEN performance creates the largest degree of separation between structured (median = 0.5991) and unstructured sequences (medians = 0.04368, 0.2625, 0.0791, 0.0789, 0.0215) (Figure 1E) as well as consistent performance to established methods (*ρ* = 0.608).

We also assessed RemuRNA, a program that compares the structural ensemble of an RNA sequence and its mutants. RemuRNA returns the KLD between the “wild-type” structure ensemble compared to the mutant-neighbor ensemble, therefore a low value indicates that the mutant secondary structure distribution is not significantly different. Using RemuRNA, there is no significant difference between the positive sequences in Dataset2 (structured median = 2.3269) and most decoy sequences (unstructured medians = 2.244, 2.246, 2.271, 2.271). Shuffled sequences do show a significant loss of neutrality compared to other data (unstructured median = 2.785) (Table 3, Additional file 1: Figure S1).

**Table 3:** **Wilcoxon rank sum determined P-values show significant difference between theneutrality of sequences**

		**3’ Flanking**		**5’ Flanking**	
**Distance metric**	**Shuffle**	**ClustalW**	**Mxscarna**	**ClustalW**	**Mxscarna**
SEN	0	2.06e-92	2.03e-92	4.99e-33	3.49e-21
Pearson’s CC	0	1.20e-108	1.92e-84	5.07e-28	1.45e-23
Bp-distance	1.14e-285	7.40e-44	7.40e-44	6.12e-19	3.75e-05
RNAmute: dot-bracket structure	2.72e-107	2.15e-10	2.15e-10	8.19e-08	1.34e-09
RNAmute: Shapiro structure	4.51e-121	8.07e-16	8.07e-16	2.43e-09	3.45e-10
RemuRNA	4.68e-132	9.99e-01	9.99e-01	9.99e-01	9.99e-01

All the neutrality measures except RemuRNA we examined are able to distinguish between structured RNAs and negative sequence datasets with statistical significance (Table 3). The neutrality of negative sequences is near the bottom of the range for each measure. In addition, shuffled sequences are particularly easy to distinguish from structured RNAs using the PCC and the SEN compared with negative data derived from flanking genomic sequence. This, combined with the fact that RemuRNA is only able to distinguish shuffled sequences from structured RNAs, suggests that column shuffled alignments may not be the most effective way to generate negative data meant to mimic natural sequences. Aligning 5’ and 3’ flanking negative data based purely on sequence (ClustalW), or using more sophisticated algorithms that consider potential structure (Mxscarna), typically does not change the results. However, the 5’-flanking negative dataset aligned using Mxscarna (5’-Mxscarna) does show significantly higher neutrality as calculated by SEN. This is caused by a poorly conserved predicted structure where each sequence only contains few predicted base pairs. This reduction in the number of base pairs in the reference structure (24.2 versus 10.9 mean base pairs per alignment for positive and 5’-Mxscarna, respectively) artificially increases SEN calculated neutrality as the potential number of base pairs that may be broken and considered deleterious is small. Despite this potential drawback in the SEN calculation, by combining an alignment based reference structure and relaxing the distance measure to consider only core structure, SEN calculated neutrality better distinguishes structured RNAs from decoy sequences than existing approaches. In addition, SEN utilizes a wider dynamic range that may allow it to have higher sensitivity. These properties are especially important for measurements that may be used as features in machine learning approaches.

### Impact of alignment quality on SEN

**Figure 2:**
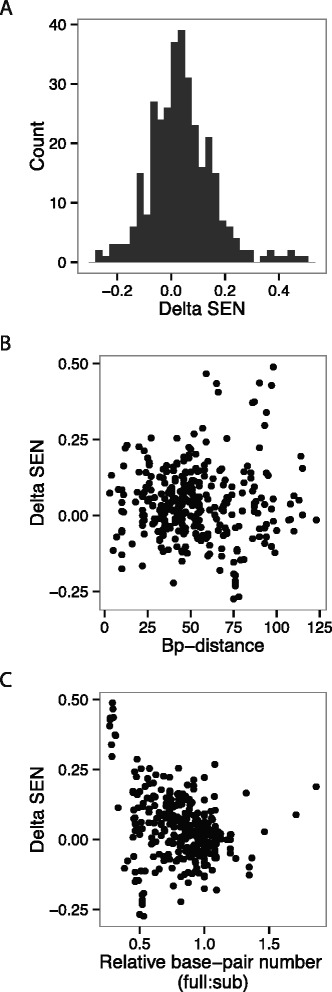
**Lower alignment quality has small impact on SEN.** The effect of alignment quality on SEN. Lower quality alignments simulated by subalignments derived from Dataset3. The delta refers to (*d* *e* *l* *t* *a*=full alignment SEN−subalignment SEN). **A)** Poorer quality alignments have a modest effect on SEN. **B)** No correlation is observed between the delta SEN and the base-pair distance between the structures derived from the full and subalignments. **C)** Large changes in relative number of base-pairs (full/subalignment) do affect SEN values.

In order to assess the effect of reduced alignment quality on SEN, we compared the difference between SEN values determined using an entire Rfam seed alignment (full alignment, Dataset2), and a subset of these sequences (subalignments, Dataset3). We observe a relatively small difference (delta) on most SEN values between the full and subalignment of the same ncRNA (Figure 2A). One common result of a lower quality alignment is altered predicted structure. To determine whether altered structure contributed to a large delta SEN, we examined the delta SEN as a function of base-pair distance between the predicted structure for the subalignment and the given structure of full alignment and found no strong correlation (Figure 2B). Since the structures for a given pair of full and sub alignments can vary in length, base-pair distance may be an imperfect comparison. Therefore, we also examined the delta SEN as a function of the ratio of the number of base pairs in the full alignment compared to the subalignment (Figure 2C). From this comparison we observe that there are a small number of subalignments that are highly impacted by using subsets of the aligned sequences. Often, these are alignments that have limited biologically relevant structure in the Rfam seed alignment, and thus may be especially prone to over prediction of structure in the subalignment. Specifically the STnc150 Hfq binding RNA (RF01402) Rfam full alignment structure has many fewer base pairs than those predicted for the subalignments.

Overall we find that SEN is robust to changes to the alignment. Most SEN values derived from lower quality alignments are within 0.2 of the full alignment (Figure 2A). The SEN calculation does not depend on perfect accuracy of the consensus structure and tolerates minor changes to the number of base pairs present. This result suggests that even alignments of relatively few sequences can be used to calculate neutrality using SEN without a large decrease in accuracy.

### Neutrality as an SVM feature

Given that most of the neutrality measures we examined exhibited a statistically significant difference between the structured and unstructured sequence, neutrality should be a highly discriminative feature in an SVM binary classifier. Because of the large separation between structured and unstructured sequence, the classification performance of SEN and PCC was predicted to be comparable to each other and higher than bp-distance. To test neutrality as a feature, we use neutrality as both an independent classifier and as part of an existing feature set for comparison with existing 6-feature SVMs [19]. First, as independent classifiers, neutrality calculated by both the SEN (Dataset2 AUC = 0.87, Dataset3 AUC = 0.903) and PCC (Dataset2 AUC = 0.864, Dataset3 AUC = 0.900) demonstrate a similar ability to correctly classify structured and unstructured sequence in all training examples regardless of sequence or structure origin (Table 4). Both of these methods significantly outperform bp-distance (Dataset2 AUC = 0.735, Dataset3 AUC = 0.766). This is likely because SEN and PCC are less stringent forms of comparison than bp-distance which equally weighs all base-pair changes, additions and disruptions.

**Table 4:** **SVM performance using neutrality as a feature**

**Data set**	**Feature(s)**	**Area under curve (AUC)**
Dataset1	6-feature set	0.918
	6-feature set + SEN	0.925
	3-feature set	0.927
	SEN	0.925
Dataset2	SEN	0.870
	PCC	0.864
	Bp-distance	0.735
Dataset3	SEN	0.903
	PCC	0.900
	Bp-distance	0.766

However, natural RNA structures do not necessarily require all base-pairs to form a biologically relevant tertiary structure. It is common to see RNA alignments containing many homologs that have pairing elements of variable length, or with mismatches within pairing elements. From biology we know that these differences in structure do not necessarily affect function. Thus, because PCC only considers effects on the overall structure, and SEN only considers changes to the core structure they more accurately reflect requirements for biological function. Consistent with our previous analysis of delta SEN, SVM performance with Dataset2 (full alignments) and Dataset3 (subalignments) is comparable.

Next, to determine whether neutrality could be used as an additional feature to improve classification of putative ncRNA alignments, we added the SEN to the 6-feature set SVM revealing a marginal improvement with SEN (Dataset1 AUC = 0.925) verse without (Dataset1 AUC = 0.918). Interestingly the SEN used in isolation as a feature has equivalent performance (Dataset1 AUC = 0.925). Using the top 3 discriminative features (Zscore of MFE structure, mean mutual information of stems, and neutrality) also had comparable performance (Dataset1 AUC = 0.927) to using SEN alone.

Overall, neutrality as an independent classifier was able to separate structured and unstructured sequences. This finding is based on the similar classification performance when using either SEN or the currently used 6-feature set (Table 4). In fact, using the most discriminating features (Zscore of the MFE structure, mean mutual information of stems and SEN) offers comparable performance indicating the remaining features are redundant. The comparable performance of neutrality with existing feature sets is likely because current methods capture aspects of neutrality: structural maintenance despite sequence mutation and thermodynamic stability. The Zscore of MFE structure measures the thermodynamic stability which is also quantified in neutrality when comparing the alignment structure to 1-mutant neighbors ensemble of structures. The structure maintenance through covarying mutation is measured using the mean mutual information of stems which neutrality encompasses as the effect of single mutations on the structure.

### Using SEN to detect structure disruption

**Figure 3:**
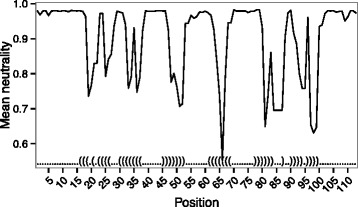
**Structure disruption generally occurs in stem regions.** Profile view of the purine riboswitch (RF00167) showing the mean neutrality at each position of all mutant neighbors at that position. The structure has been overlaid onto the graph. Mutations in the stems show larger structure disruption whereas mutations which occur in the single stranded regions do not significantly affect structure.

One objective of many neutrality measures is to predict which bases are most disruptive to structure [3,5,37]. To evaluate whether SEN can be used to predict such bases, we sampled multiple sequences from our training set and interrogated the effect of position specific mutations on the calculated neutrality. Though the neutrality profiles by position are not identical for all sequences, the neutrality predicted by SEN has consistent performance across multiple sequences drawn from the same alignment. In agreement with previous observations [38,51], mutations to bases in structured regions (Figure 3) are more likely to be disruptive. The most disruptive mutations occur at the edges of stems. Mutations in the middle of stems appear to create either bulges or internal loops which have a small effect on the neutrality. Mutations in the loop regions also had little effect on the structure.

**Figure 4:**
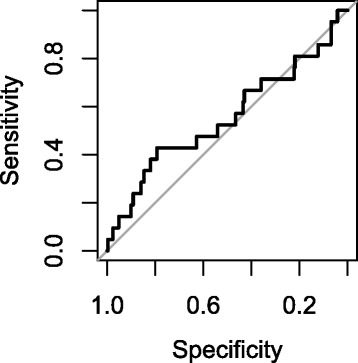
**Ability to predict which base mutations disrupt structure.** The receiver operator characteristic (ROC) curve shows the performance of SEN to correctly call structure disrupting mutations compared to random guessing (diagonal line). The line reveals SEN performs better than random.

To assess the accuracy of predicted structure disrupting mutations, we compared our predictions to experimental data obtained on the purine riboswitch using 2D SHAPE (Selective 2’-hydroxyl acylation analyzed by primer extension) [52]. Like evaluating neutrality using 1-mutant neighbors, 2D SHAPE interrogates the changes in RNA structure when making single mutations to an RNA sequence. To compare our predictions to the 2D SHAPE data, the reported change in base reactivity was converted to the expected structure disruption coefficient (eSDC) using $(1-PCC)*\sqrt {L}$ [40]. The top 50% of eSDC values are considered to be “structure disrupting”. Logistic regression using SEN to predict structure disruption indicates that predicting which bases disrupt structure continues to be very difficult (AUC = 0.55) (Figure 4).

Current methods rely on RNA folding algorithms to predict which nucleotides can potentially be structure disrupting. Incorporating the structure ensemble does improve prediction accuracy [40] but such methods fundamentally still have poor performance. The similar predictions of both SEN and current methods to detect structure disruption is likely due to the use of the same thermodynamic model for RNA folding that cannot fully encompass three-dimensional interactions, which results in similar prediction accuracy. However, the inability of SEN to make accurate predictions could also be due to the limited data on structure disrupting bases derived from 2D SHAPE. Because a vast majority of positions have small impacts on structure, it is very difficult to establish the eSDC threshold at which the structure is disrupted. Furthermore, if the eSDC threshold is too high, then there is very little data available to build regression or machine learning models.

### SEN detects mutational robustness

**Figure 5:**
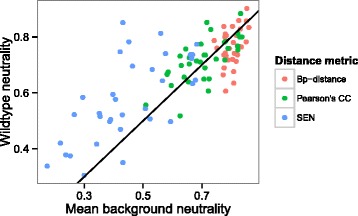
**Mean alignment neutrality organized by distance metrics.** The line represents wildtype sequence neutrality equal to mean background neutrality. If the wildtype sequence neutrality is higher than the mean background neutrality, the sequence is considered robust. To reduce the number of points, only the mean sequence neutrality for an alignment is compared against the average of the mean background neutrality. Plotting individual sequence neutrality reveals a similar trend (Additional file 1: Figure S2). The SEN better detects mutational robustness of these sequences compared to PCC or bp-distance.

Finally, we use SEN to calculate the mutational robustness of positive sequences in our data sets. Robustness is defined as the ability of a sequence to maintain its structure despite perturbations to the sequence. The sequence is considered mutationally robust when its neutrality is greater than the mean background neutrality. Using SEN as a distance measure detects 74.9% of the sequences in Dataset2 as being mutationally robust (Table 5). In comparison, using PCC (58.8%) or bp-distance (40.5%) detected fewer robust sequences. The background neutrality calculated by PCC and bp-distance is relatively high compared to the SEN background neutrality and likely contributes to the ability of distance measures to detect mutational robustness (Figure 5, Additional file 1: Figure S2).

**Table 5:** **Fraction of robust sequences**

Bp-distance	0.405
PCC	0.588
SEN	0.749

Despite the equivalent classification performance of PCC and SEN in the SVM, PCC has reduced ability to detect mutational robustness. The PCC calculation involves converting the structure into a binary vector; therefore, the base pairing information is removed and only the base-pairing status remains. By removing this information, the PCC potentially has difficulty differentiating similar distributions of 0’s and 1’s which could represent different structures. Bp-distance had difficulty detecting mutational robustness in the data, likely due to the high stringency of the neutrality measure. Thus, existing commonly used measures of neutrality, normalized base-pair distance and PCC have potentially decreased accuracy for opposite reasons. The ability of SEN to detect mutational robustness in ncRNA regulators can likely be attributed to the hybrid nature of the calculation which still considers individual base pairs but is only concerned with the maintaining the core structure and not with additional base pairs added by in 1-mutant neighbor.

### SEN run time

SEN relies on the sampling of suboptimal structures from the ensemble of secondary structures. The run time is directly proportional the number of sampled suboptimal structures and thus slower than traditional methods like bp-distance. However, the calculation for each sample structure is identical so SEN calculations have been implemented to run in parallel, which can significantly reduce the run time. Code for calculating SEN is available at: https://github.com/ship561/sampled-ensemble-neutrality.

## Conclusions

In this work, we show that RNA sequence neutrality is an effective feature for machine learning approaches to classify structured RNAs from various decoy sequences. We find that the most accurate classification occurs for neutrality measures that consider the ensemble of possible RNA structures rather than just the minimum free energy structure (PCC or SEN). Furthermore, neutrality used as the sole classifying feature is nearly as effective as existing SVMs [17,19] indicating that current SVM features capture aspects of mutational robustness.

During the course of this work, we developed a novel measure of RNA sequence neutrality, the structural ensemble neutrality (SEN). The SEN differs from existing measures of neutrality in that it directly addresses several potential limitations. First, as a reference structure for neutrality calculation, SEN utilizes a consensus structure determined from an alignment of putative homologous sequences rather than an MFE structure, increasing the likelihood of utilizing a biologically relevant reference. Second, to assess the structure of the 1-mutant neighbors SEN considers not a single structure, but samples from the ensemble of potential low-energy structures. Finally, rather than consider all deviations from the reference structure equally deleterious, SEN only counts base pairs that are disrupted in the structure of the mutant sequence. This property renders SEN relatively robust to incomplete data that often degrades the quality of the predicted structure. The SEN is highly correlated with existing measures of neutrality (Table 2), but shows improved separation of structured and unstructured sequences in our data sets compared to these measures (Figure 1). While SEN’s underlying model predicts structure disrupting mutations to occur in stems, this model does not completely explain experimental data (Figure 4) indicating there are other variables such as potential tertiary contacts to consider in such determinations. However, SEN does indicate that many of regulatory RNA structures in bacteria are mutationally robust (Figure 5).

## Additional file

Additional file 1
**Supplemental data.** The file is in a PDF format. It contains Table S1 and Figures S1 and S2. Figure S1 shows the distribution of neutrality values returned by RemuRNA on Dataset2 compared with various decoy datasets. Figure S2 shows the robustness of sequences in Dataset2 organized by distance metric. Table S1 is a table listing the Rfam families used as positive alignments (Dataset2).

## Abbreviations

ncRNA: Non-coding RNA
MFE: Minimum free energy
SEN: Structure ensemble neutrality
PCC: Pearson’s correlation coefficient
KLD: Kullback-Leibler divergence
MSA: Multiple sequence alignment
Bp-distance: normalized base-pair distance
db: dot-bracket
ROC: Receiver operating characteristic
