# Supplementary material for: Sampled ensemble neutrality as a feature to classify potential structured RNAs
Source: BMC Genomics. 2015 Feb 5;16(1):35. doi: 10.1186/s12864-014-1203-8 (PMC4333902; doi:10.1186/s12864-014-1203-8)
Supplement: Additional file 1 — Supplemental data. The file is in a PDF format. It contains Table S1 and Figures S1 and S2. Figure S1 shows the distribution of neutrality values returned by RemuRNA on Dataset2 compared with various decoy datasets. Figure S2 shows the robustness of sequences in Dataset2 organized by distance metric. Table S1 is a table listing the Rfam families used as positive alignments (Dataset2). [file 12864_2014_1203_MOESM1_ESM.pdf]

Supplemental tables and figures for Pei *et. al.*

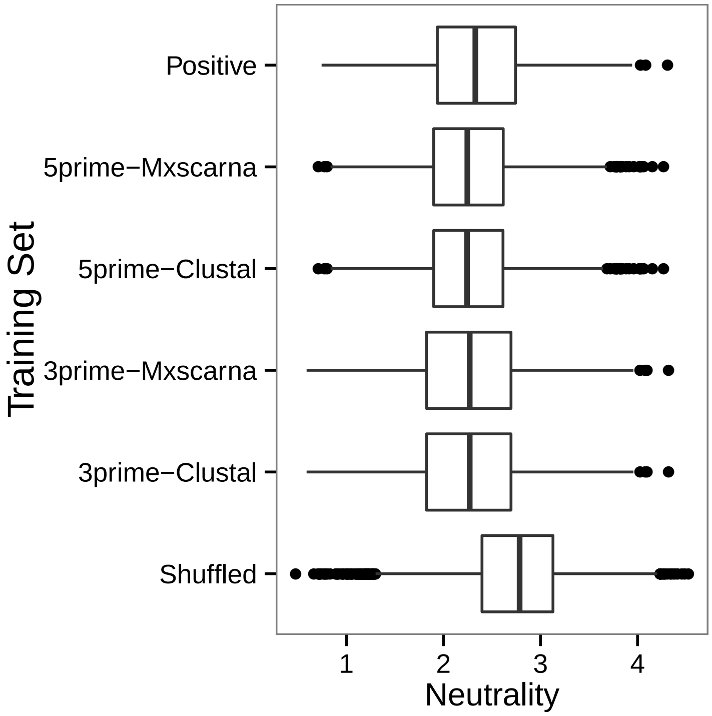

Figure S1: RemuRNA distinguishes shuffled data but not other decoy sequences from positive sequences in Dataset2. Distribution of neutrality values from Dataset2 were compared using RemuRNA. The 3' and 5' flanking region used for negatives are referred to as 3prime and 5prime, respectively. There is no difference in the positive test set compared to the 5' and 3' negatives. There is a difference between the positive and shuffled alignments.

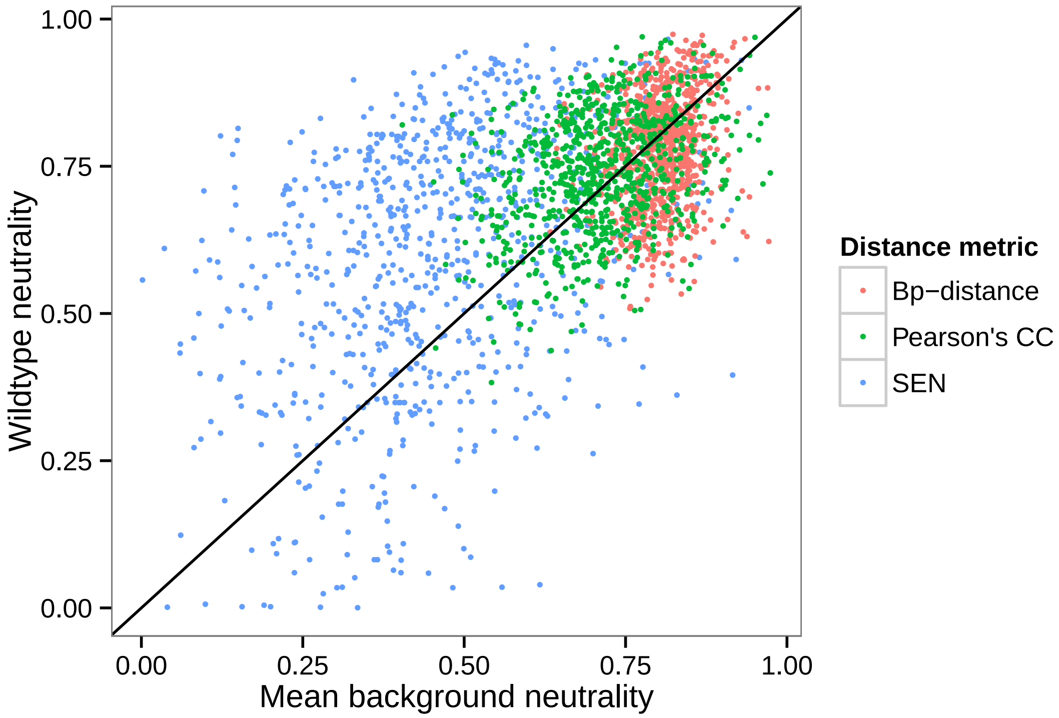

Figure S2: SEN calls more sequences robust than other distance metrics. The line represents wildtype sequence neutrality equal to mean background neutrality. If the wildtype sequence neutrality is higher than the mean background neutrality, the sequence is considered robust. Each point represents a sequence in the Dataset2 and it is compared against the average of the mean background neutrality. The SEN better detects mutational robustness of these sequences compared to PCC or bp-distance.

Table S1: List of *cis*-regulatory RNAs in Dataset2 and the number of sequences in each alignment

Rfam ID	Name	Number of sequences
RF00050	FMN riboswitch (RFN element)	112
RF00057	RyhB RNA	26
RF00059	TPP riboswitch (THI element)	86
RF00114	Ribosomal S15 leader	62
RF00167	Purine riboswitch	106
RF00168	Lysine riboswitch	36
RF00234	glmS glucosamine-6-phosphate activated ribozyme	16
RF00379	ydaO/yuaA leader	93
RF00380	ykoK leader	78
RF00442	ykkC-yxkD leader	80
RF00504	Glycine riboswitch	40
RF00506	Threonine operon leader	17
RF00514	Histidine operon leader	22
RF00515	PyrR binding site	48
RF00522	PreQ1 riboswitch	31
RF00555	Ribosomal protein L13 leader	26
RF00557	Ribosomal protein L10 leader	84
RF00558	Ribosomal protein L20 leader	36
RF00559	Ribosomal protein L21 leader	36
RF01051	GEMM cis-regulatory element	104
RF01055	Moco (molybdenum cofactor) riboswitch	123
RF01057	S-adenosyl-L-homocysteine riboswitch	37
RF01070	SucA RNA motif	26
RF01385	isrA Hfq binding RNA	8
RF01402	STnc150 Hfq binding RNA	9
RF01482	AdoCbl riboswitch	5
RF01510	M. florum riboswitch	2
RF01692	Bacteroidete tryptophan peptide leader RNA	13
RF01693	Bacteroidales-1 RNA	7
RF01694	Bacteroides-1 RNA	8
RF01727	SAM/SAH riboswitch	12
RF01767	SMK box translational riboswitch	11
RF01769	Enterobacteria greA leader	19
RF01793	ffh sRNA	36
RF01826	SAM-V riboswitch	3
Total		1458
